# Supplementary material for: Actomyosin-dependent dynamic spatial patterns of cytoskeletal components drive mesoscale podosome organization
Source: Nat Commun. 2016 Oct 10;7:13127. doi: 10.1038/ncomms13127 (PMC5062568; doi:10.1038/ncomms13127)
Supplement: Supplementary Information — Supplementary Figures 1-8, Supplementary Methods and Supplementary References. [file ncomms13127-s1.pdf]

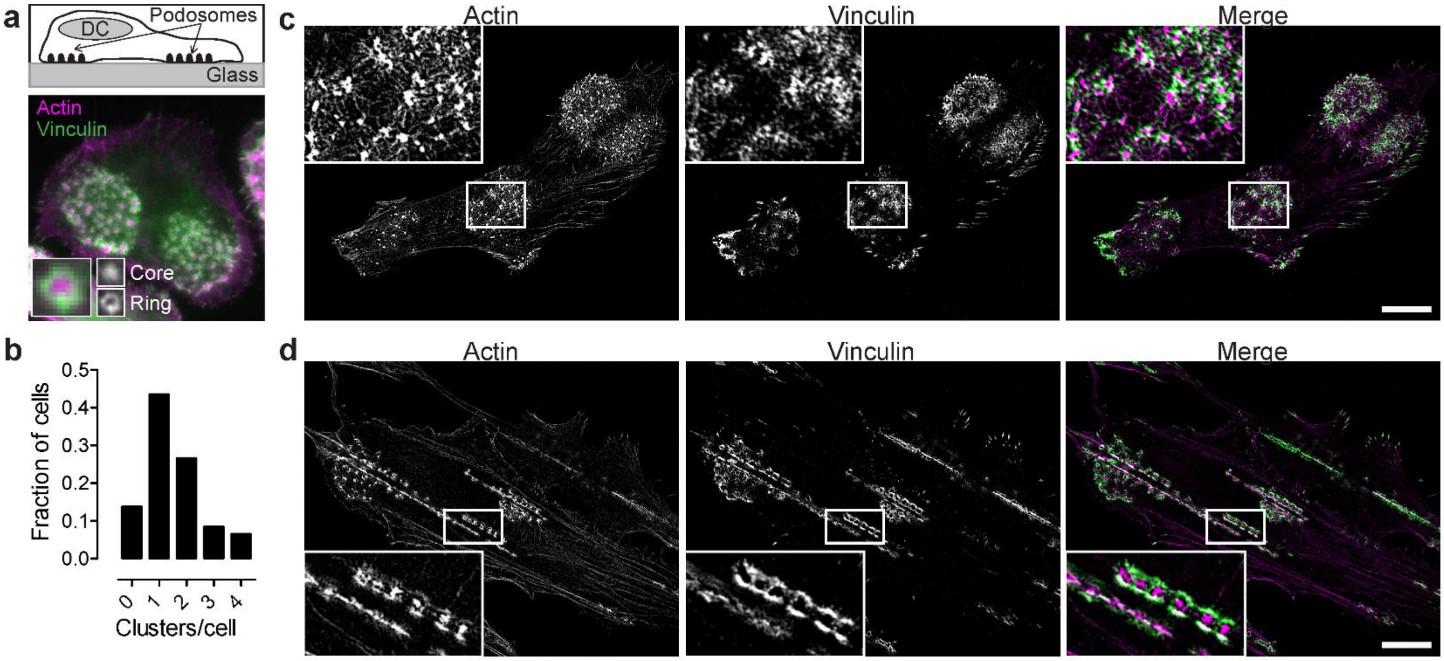

Supplementary Figure 1. Spatial organization of podosome clusters in dendritic cells.

(**a**) DCs adherent to a glass coverslip (as depicted in the cartoon) were fixed, permeabilized and labelled for actin (magenta) and vinculin (green) by fluorescent phalloidin and anti-vinculin mAb, respectively. Samples were mounted in Mowiol and imaged by epi-fluorescence microscopy. One representative image is shown with three small insets depicting one individual podosome (merged image) as well as the actin (core) and vinculin (ring) signals. (**b**) Quantification of the number of podosome clusters per cell as determined by analysing images of 50 fixed DCs from 2 experiments. DCs adhering to flat (**c**) or scratched (**d**) coverslips were fixed, permeabilized and labelled for actin (magenta) and vinculin (green) by phalloidin and anti-vinculin mAb, respectively. Samples were mounted in mowiol and imaged by SIM. Scale bars 10 µm.

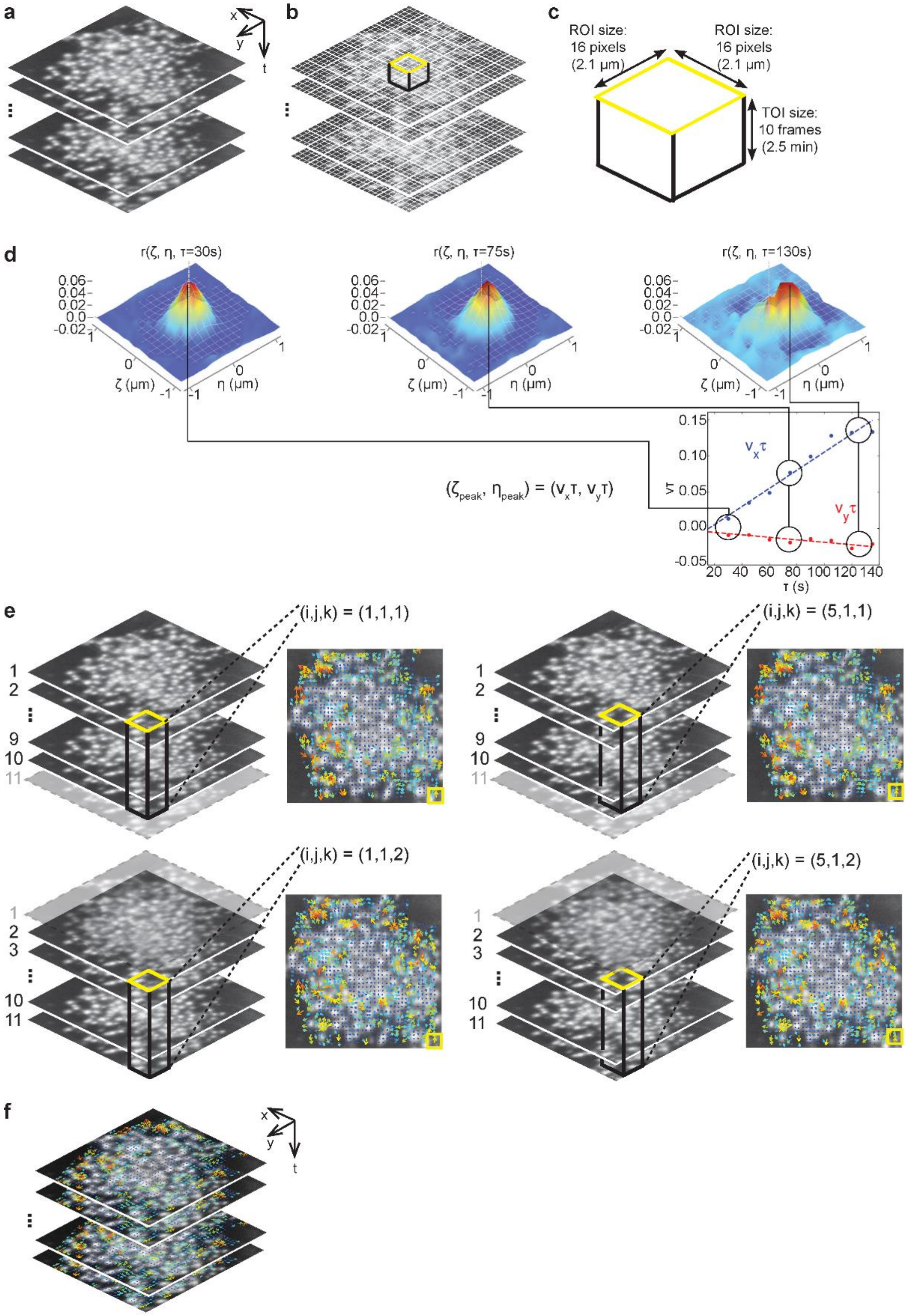

Supplementary Figure 2. Schematic overview of twSTICS analysis

(**a**) Time series of fluorescently tagged podosome components with a frame time of 15 seconds are acquired. (**b,c**) Each image is divided into 16x16 pixel regions of interest (ROIs) (2.24 µm x 2.24 µm) and adjacent ROIs are shifted four pixels to map the entire field of view. The time series is divided into 10 frame sized time of interest (TOIs) (2.5 min) and adjacent TOIs are shifted one frame to cover the entire image series. (**d**) For each ROI-TOI of 16 pixels by 16 pixels by 10 frames the spatiotemporal autocorrelation is calculated for all possible image pairs. The shift of the correlation peak is depicted at time lag τ=30, 75 and 135s. From the shift of the correlation peak as a function of time lag, protein velocity is calculated. (**e**) For each ROI the velocity vector is plotted at the corresponding position on the immobile filtered version of the original image series. The direction of the vector represents the direction of molecular motion whereas both the size and colour of the velocity vector represent the magnitude. (**f**) The STICS analysis is repeated for each TOI resulting in a time series of evolving vector maps. The yellow square is drawn to scale in each panel. More details on the twSTICS can be found in the supplementary methods.

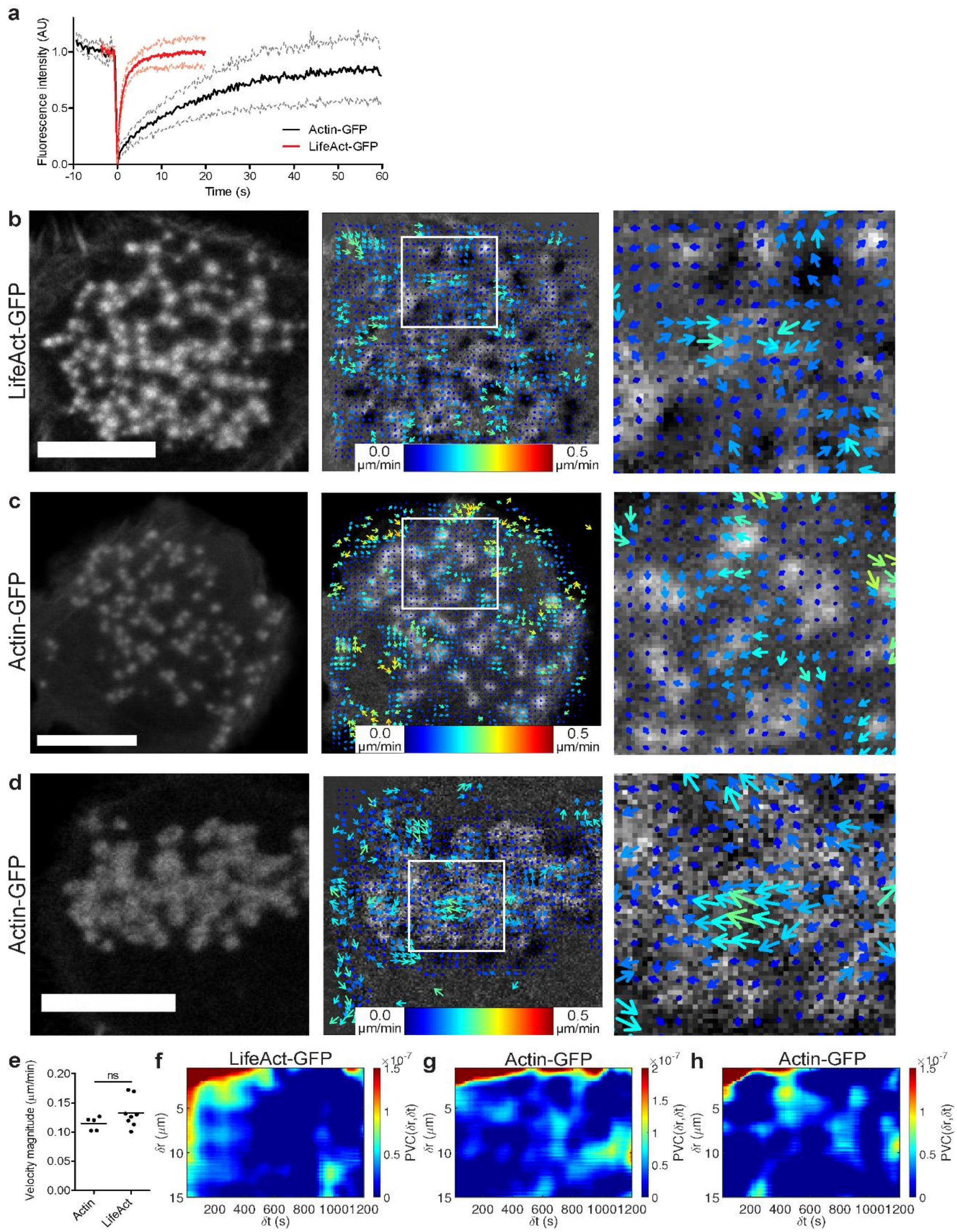

Supplementary Figure 3. LifeAct-GFP can report actin flux in podosome clusters

DCs were transfected with actin-GFP (black) or LifeAct-GFP (red) and FRAP analysis was performed (**a**). 12 cells were analysed for each protein and average recovery curves are shown. Fluorescence intensity in bleach ROI is set to zero post-bleach and max intensity is normalized to one. Dotted lines represent standard deviation. DCs were transfected with LifeAct-GFP (**b**) or actin-GFP (**c,d**) and seeded in a glass bottom dish. Imaging was performed at a confocal microscope with 15 second frame intervals. Time series (100 frames) were subjected to twSTICS analysis and results are shown as vector maps in which the arrows indicate direction of flow and both the size and colour coding are representative of the flow magnitude. Shown are the original images and corresponding vector maps plotted onto the immobile filtered version of the image (**b**,**d**) or 10 frame moving average (**c**). Quantification of flow magnitudes is shown in (**e**), where each dot represents a single cell and the line shows mean. Means were not significantly different (paired student t-test, two-tailed, p=0.1668). PVC plots show the spatial and temporal scales of vector correlation for LifeAct-GFP (**f**) and actin-GFP (**g**,**h**). Scale bars represent 10 µm, ns = not significant.

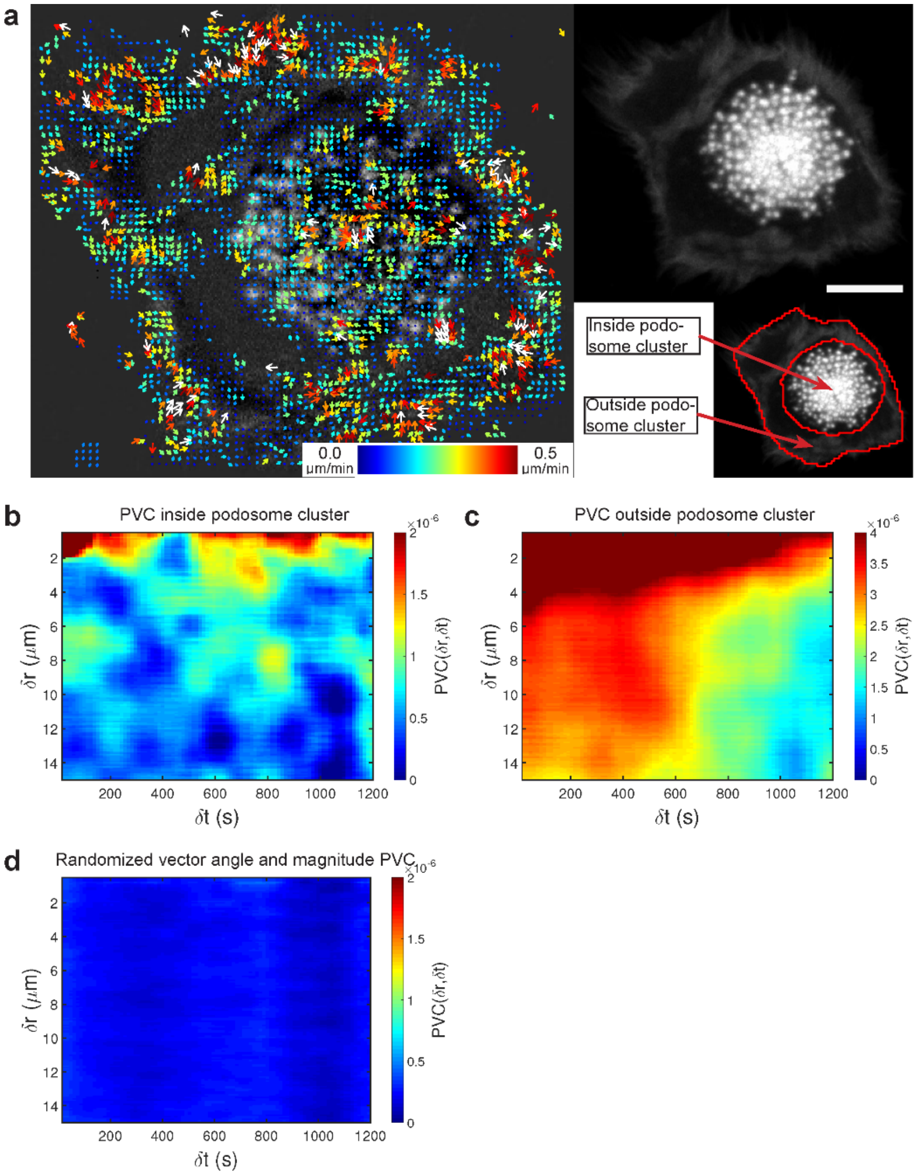

Supplementary Figure 4. Pair Vector Correlation controls

DCs were transfected with LifeAct-RFP and seeded in a glass bottom dish. Imaging was performed at a confocal microscope with 15 second frame intervals. Time series (100 frames) was subjected to twSTICS analysis, results are shown as vector maps in which the arrows indicate direction of flow and both the size and colour coding are representative of the flow magnitude (**a**, left panel). Vector maps are plotted onto the immobile filtered version of the image. Top right panel shows a 10 frame moving average image from the time series. Bottom right panel shows regions considered as inside and outside the podosome cluster. PVC plots were calculated for all vectors inside (**b**) and outside (**c**) the podosome cluster. Also, all vectors were randomly assigned an angle and magnitude, taken from the real distribution of angles and magnitudes. Next, PVC was calculated for these randomized vectors within the whole cell (**d**). Scale bars represent 10 µm.

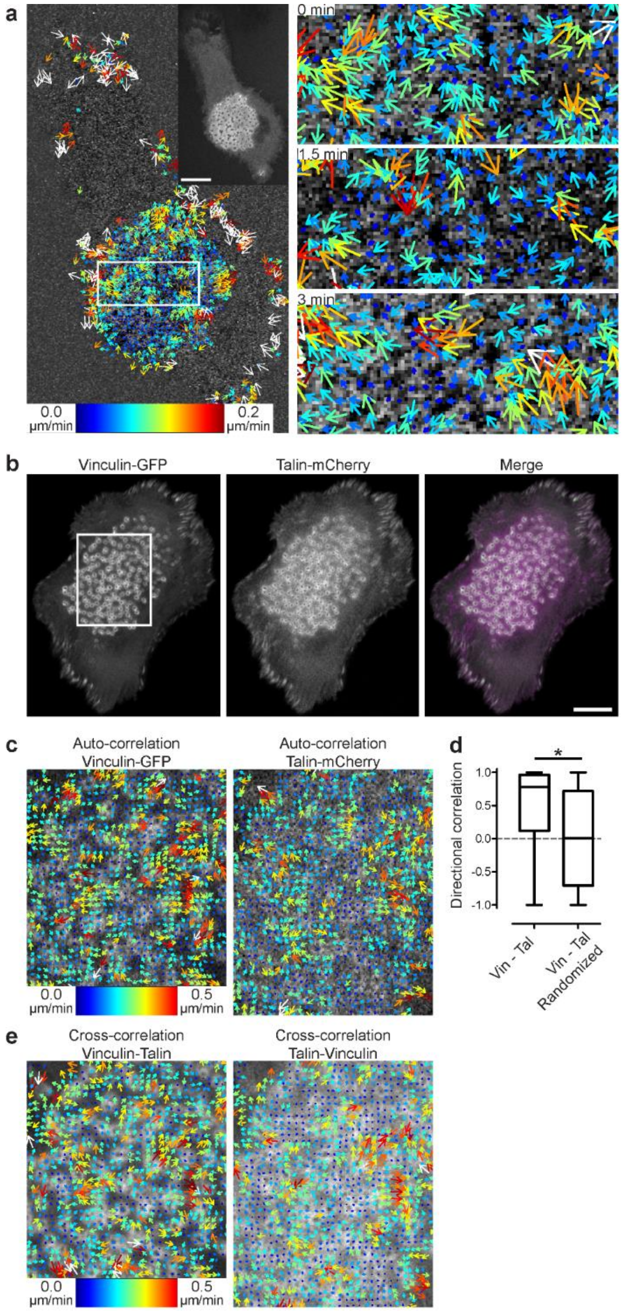

Supplementary Figure 5. TwSTICS shows talin flux in podosome clusters and STICCS shows cross correlation between vinculin and talin in podosome clusters

DCs were transfected with talin-GFP and seeded in a glass bottom dish. Imaging was performed at a confocal microscope with 15 second frame intervals. Time series (100 frames) were subjected to twSTICS analysis. Results are shown as vector maps in which the arrows indicate direction of flow and both the size and color coding are representative of the flow magnitude (**a**). Vector maps are plotted onto the immobile filtered version of the images. A 10 frame moving average corresponding to the STICS window is shown in the upper right inset. On the right zoomed in images of the boxed region are shown for three different STICS time windows. DCs were co-transfected with vinculin-GFP and talin-mCherry and seeded in a glass bottom dish. Imaging was performed at a confocal microscope with 15 second frame intervals. 10 frame moving average images are shown in (**b**). Time series for both channels (100 frames) were subjected to STICS analysis, results are shown as vector maps in which the arrows indicate direction of flow and both the size and colour coding are representative of the flow magnitude (**c**). Vector maps are plotted onto the immobile filtered version of the image. Directional correlation was calculated between vectors measured by STICS in two different channels as the cosine of the angle between vectors with the same spatial and temporal lag. The distribution is compared to the directional correlation of one original channel with the other channel containing original vectors which are all randomly assigned to a spatiotemporal position (**d**). Time series were also subjected to spatio-temporal image cross-correlation spectroscopy (STICCS) analysis (**e**). STICCS vector maps are shown for analysis flow of talin relative to vinculin (left panel) and vice versa (right panel). Asterisks indicated statistically significant differences (Mann-Whitney U test, two-tailed, $p<0.001$). Scale bars represents 10 μm.

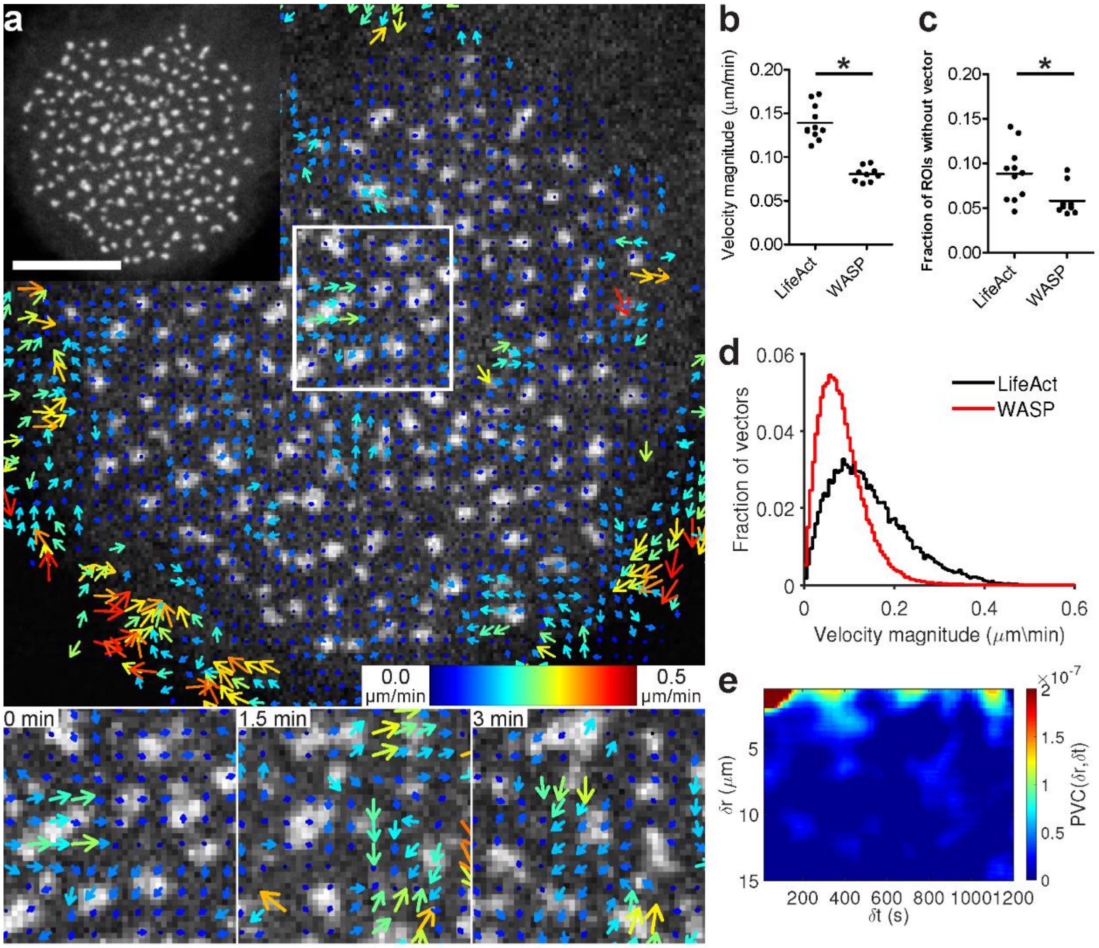

Supplementary Figure 6. twSTICS analysis of WASP-GFP

DCs were transfected with WASP-GFP and seeded in a glass bottom dish. Imaging was performed at a confocal microscope with 15 second frame intervals. One representative movie out of nine recorded from two independent experiments is shown. Scale bars represents 10 µm. Time series (100 frames) were subjected to twSTICS (**a**). Results are shown as vector map in which the arrows indicate direction of flow and both the size and colour coding are representative of the flow magnitude. The vector map is plotted onto the immobile filtered version of the images. A 10 frame moving average corresponding to the STICS window is shown in the upper left inset. At the bottom, zoomed in images of the boxed region are shown for three different STICS time windows. Mean STICS velocity magnitudes (**b**) and fraction of ROIs (**c**) without vector for WASP-GFP within podosome clusters was compared with LifeAct-GFP transfected DCs (not shown in this figure) from the same donors. Each dot represents a single podosome cluster in a different cell, the line shows the mean, and asterisks indicate statistically significant differences (student t test, unpaired, two-tailed, (**b**) p=1.62e-7, (**c**) p=0.0159). By pooling all cells, histograms of velocity magnitude values for WASP-GFP and LifeAct-GFP are obtained (**d**). PVC plot (**e**) shows the spatial and temporal scales of vector correlation for WASP-GFP.

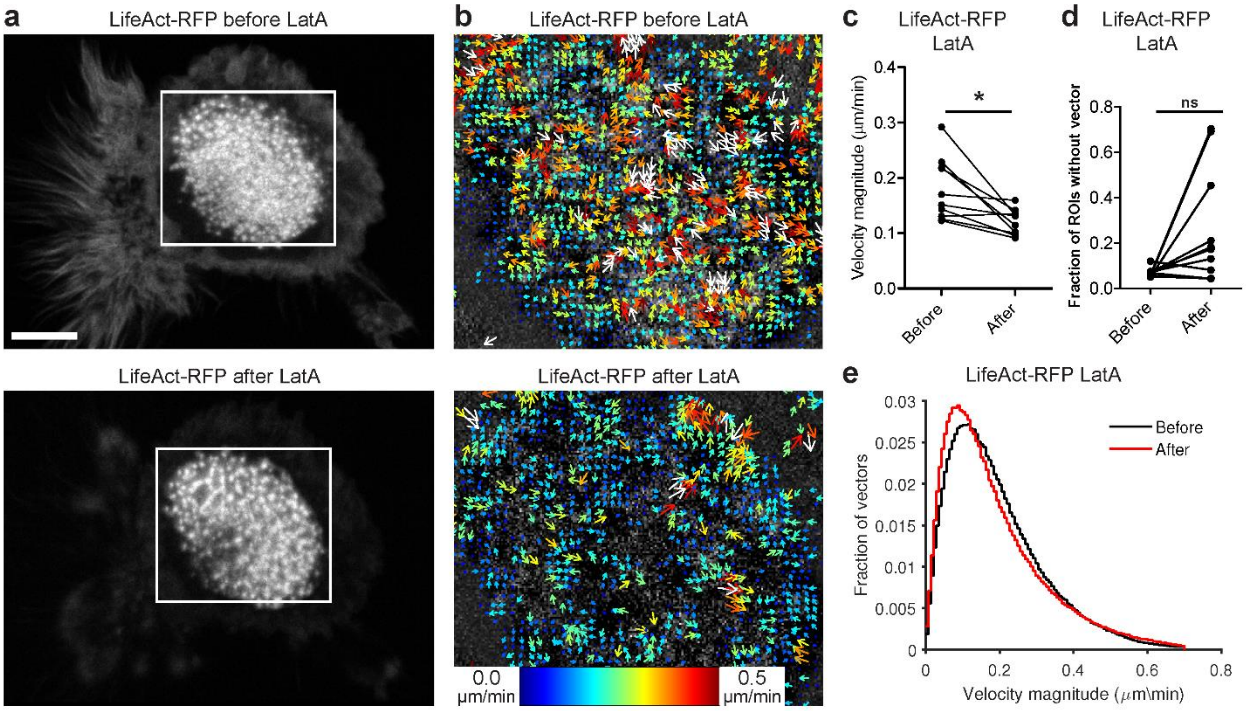

Supplementary Figure 7. Latrunculin A disrupts actin flux in podosome clusters

DCs were transfected with LifeAct-GFP and seeded in a glass bottom dish. Imaging was performed at a confocal microscope with 15 second frame intervals, after 50 frames 0.5 µM LatA was added and imaging was continued up to 100 frames. 10 frame moving average images are shown before and after the addition of LatA (**a**). Time series were subjected to twSTICS analysis. Images and corresponding vector maps plotted onto the immobile filtered version of the image are shown before and after addition of LatA (**b**). One representative movie out of 11 cells imaged in two separate experiments is shown. The arrows indicate direction of flow and both the size and colour coding are representative of the flow magnitude. Quantification of per cell flow magnitudes (**c**) and fraction of empty ROIs (**d**) are shown before and after addition of LatA. Each dot represents a single podosome cluster in a different cell, lines connect the same cells before and after treatment, and asterisk indicates statistically significant difference (student t test, paired, two-tailed, (**c**) p=0.0072, (**d**) p=0.1437). Pooling all the cells used for panels **c** and **d**, histograms of velocity magnitudes (**e**) are shown before and after addition of LatA. Scale bars represent 10 µm, ns = not significant.

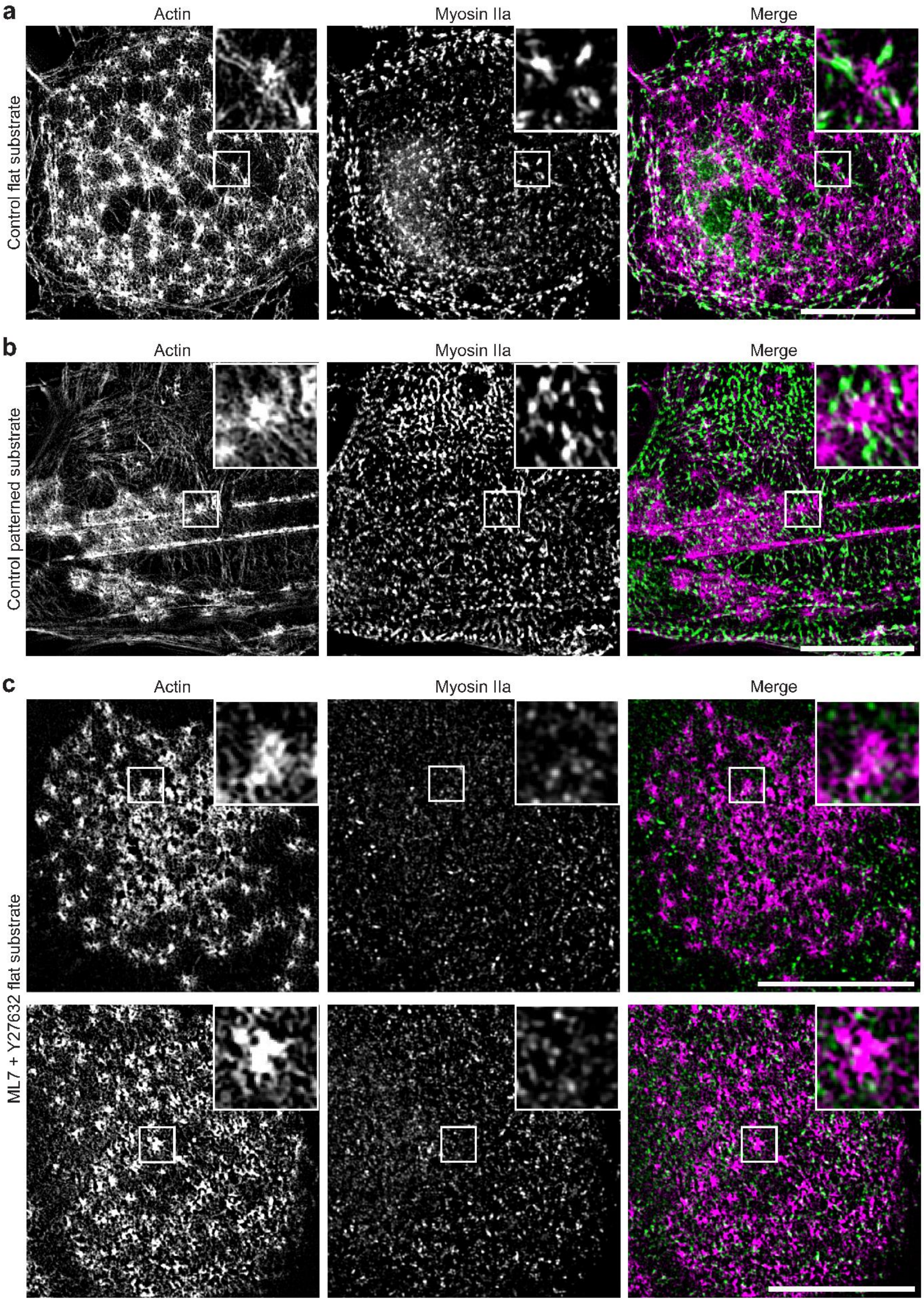

Supplementary Figure 8. SIM images of myosin IIA and actin at podosome clusters.

DCs adhering to flat (**a,c**) or scratched (**b**) coverslips, were left untreated (**a,b**) or treated with a cocktail of myosin IIA inhibitors (**c**, ML7 10 µM and Y27632 20 µM) for 60 min. Cells were fixed, permeabilized and labelled for actin (magenta) and myosin IIA (green) by phalloidin and anti-myosin IIA mAb, respectively. Samples were mounted in mowiol and imaged by SIM. Scale bars 10 µm.

Supplementary Methods

Time-window STICS analysis

We performed sliding time-window (tw) STICS on CLSM time series of dendritic cells expressing fluorescent protein conjugates, acquired with a 15 s time interval between frames (Supplementary Fig. 1a). STICS was originally developed as a technique [1, 2, 3] to measure vector maps of the directed transport or flow of proteins inside living cells and twSTICS is a simple extension which measures the time evolution of the vector maps over shorter time windows. For these studies, each 16x16 pixels region of interest (ROI: yellow square in Supplementary Fig. 1b,c) is shifted in x and y by 4 pixels, in order to spatially oversample adjacent ROIs. STICS relies on calculating the spatio-temporal pixel intensity fluctuation correlation function in parallel from ROIs in images of a time series which is then fit with a 2D Gaussian function for each time lag. The Gaussian centroid peak is tracked in time and fit to output vector maps of the flow of fluorescently tagged molecules (Supplementary Fig. 1d). Decreasing the ROI size to smaller areas results in noisy correlation functions in which the fitting of the centroid peak becomes unreliable.

twSTICS involves narrowing the time window of interest (TOI) to a small number of image frames, performing STICS analysis on this smaller ROI-TOI image substack (Supplementary Fig. 1b,c), and iteratively advancing the TOI window one frame at a time through the series in order to calculate a time evolving set of STICS vector maps. This results in a smooth variation in the vector field map when STICS analysis is carried out in parallel on the spatially oversampled ROIs for a set TOI window (Supplementary Fig. 1e). Moreover, the spatial oversampling permits a magnitude and direction comparison between adjacent vectors which is used for filtering the noisy outlier vectors when mapping real flows (see details below).

The TOI windows are then shifted by a single image frame in time and the STICS analysis is performed again on the full field of spatially oversampled ROIs to yield STICS vector map at the new time point (Supplementary Fig. 1e). The time shifting of the ROI-TOI substacks by a single frame followed by parallel STICS analysis on the ROIs is repeated iteratively until the sliding window analysis reaches the end of the CLSM image stack. The output from twSTICS is a time evolving movie of STICS vector maps which can be further analysed qualitatively or quantitatively (Supplementary Fig. 1f).

Time-window Spatio-temporal cross correlation spectroscopy

twSTICCS was performed to investigate cross correlation between vinculin and talin. twSTICCS was carried out as described for twSTICS above and in the Supplementary Information with a slight variation in the calculation of the spatial-temporal correlation function. For twSTICCS, a spatio-temporal cross correlation function (CCF) was calculated between ROI-TOI substacks for two detection channels from simultaneously acquired vinculin and talin image time series as described earlier[3]. The CCF was calculated in two ways: for talin relative to vinculin and for vinculin relative to talin, which always yielded similar results. TwSTICCS for actin and the various ring components is hampered by the distinct locations of actin and the other components within individual podosomes. In this case, the zero time lag Image Cross-Correlation Function (ICCF) function is donut shaped and not peaked. It is therefore challenging to infer spatiotemporal dynamics from the changing of ICCF, since peak fitting will not give accurate results. Adaptation of the ICCF fitting in the STICCS method could provide more detail on direct interactions and the order of events.

Immobile population removal

Prior to twSTICS, the entire image series is processed in order to remove the immobile/very slowly moving populations as has been described previously [2]. An immobile population within the image series will contribute a static correlation peak in the STICS analysis that can mask contributions from dynamic components and skew the fitting for dynamic components resulting in systematic underestimates of flow velocities. The filtering of the immobile population is done in the time frequency space, where the lowest (time) frequency component of each pixel stack in the image series, is set to zero, and the filtered sequence is transformed back to the time domain. The filtered intensity of each pixel is given by:

$$i'(x,y,t) = F_f^{-1}\left\{F_t\{i(x,y,t)\} \times H_{\frac{1}{T}}(f)\right\} \qquad [1]$$

where F_a and F_a^{-1} are the forward and inverse Fourier transforms with respect to the variable a, $H_{1/T}(f)$ is the Heaviside step function, which is 0 for $f < 1/T$ and 1 for $f > 1/T$, f is the pixel frequency in time, and T is the total acquisition time of the image time series. For twSTICS, we apply the immobile filtering to the whole image time series, and not on separate ROI-TOIs in order to remove only the lowest frequency components and effectively filtering the global immobile population present in the time series.

STICS analysis of an immobile filtered ROI-TOI substack

Following the immobile filtering, the discrete STICS correlation function is calculated for each ROI-TOI substack using:

$$r(\zeta,\eta,\tau) = \frac{1}{T-\tau}\sum_{t=1}^{T-\tau}\frac{1}{M-\zeta}\sum_{x=1}^{M-\zeta}\frac{1}{N-\eta}\sum_{y=1}^{N-\eta}\frac{\delta i(x,y,t)\delta i(x+\zeta,y+\eta,t+\tau)}{\langle i\rangle_t\langle i\rangle_{t+\tau}} \qquad [2]$$

where (M,N,T) are dimensions of ROI-TOI in pixels, (Supplementary Fig. 1c) (16,16,10) in these studies, the integer spatio-temporal lag variable (ζ,η,τ) represent pixel shifts in space and time for the correlation calculation, the average pixel intensity of the ROI at t and $t + \tau$ are labelled $\langle i \rangle_t$ and $\langle i \rangle_t + \tau$ respectively, while the intensity fluctuation of the pixel at position (x,y,t) is $\delta i(x,y,t) = i(x,y,t) - \langle i \rangle_t$. Equation 2 takes into account the intensity fluctuations of all pairs of pixels separated by spatio-temporal lags (ζ,η,τ). In practice it is computationally more efficient to calculate the correlation function via a fast Fourier transform (FFT) and complex conjugate multiplied in k-space, followed by inverse fast Fourier transformation (IFFT) to yield:

$$r(\zeta,\eta,\tau) = \frac{1}{T-\tau}\sum_{t=1}^{T-\tau}\frac{F_{x,y}^{-1}\{\tilde{\imath}(k_x,k_y,t)\tilde{\imath}^*(k_x,k_y,t+\tau)\}}{\langle i\rangle_t\langle i\rangle_{t+\tau}} \quad [3]$$

where $\tilde{\imath}(k_x,k_y,t)$ is the FFT of image $i(x\ y,t)$ defined in k-space by the wave vectors $k = (k_x,k_y)$ and * is used to denote the complex conjugate of the Fourier transformed image.

Fitting the STICS correlation function

At zero time lag the STICS correlation function from image series will have a 2D Gaussian shape with peak centred at zero spatial lags $(\zeta,\eta = 0)$. Depending the transport properties of the fluorescent molecules, the correlation function will either translate (flow only), spread isotropically (diffusion only) or translate and spread isotropically (flow and diffusion simultaneously) in spatial lag coordinate space as the time lag increases. Full details on STIC(C)S correlation function decays related transport were described previously[2, 3]. The general function used for fitting the STICS correlation function is a time translating and spreading 2D Gaussian function:

$$r(\zeta,\eta,\tau) = A(\tau)\times exp\left\{\frac{-\omega_y^2(\tau)(\zeta - v_x\tau)^2 - \omega_x^2(\tau)\left(\eta - v_y\tau\right)^2}{4\omega_w^2(\tau)\omega_y^2(\tau)}\right\} \quad [4]$$

where $A(\tau)$ and $\omega_{x,y}(\tau)$ are time dependent amplitude and e^{-2} radii in x and y directions, respectively. The time dependent peak position of the correlation function is specified by $(v_x\tau, v_y\tau)$. Thus the fit peak positions vs. temporal lag τ can be used to calculate the vector of the velocity field for the given ROI-TOI. In the case of purely flowing particles, $A(\tau)$ and $\omega_{x,y}(\tau)$ will be independent of τ (i.e. constant), assuming no photobleaching. On the other hand, for simultaneously flowing and diffusing particles, as in the case of a biased random walk, the amplitude $A(\tau)$ will decay with τ while square of the radii of the correlation function, $\omega_{x,y}(\tau)$ will grow linearly with τ. These can be used to calculate the diffusion coefficient of particles. In the current work only the correlation peak centroid position $(\zeta_{peak}\ \eta_{peak})$ is fit as a function of lag time · in order to determine a vector for particle flow velocity for each ROI-TOI substack as the image frame sampling rate was not high enough to capture diffusion of the proteins (Supplementary Fig. S1d).

Filtering the outlier vectors

The twSTICS code implements several tests to verify the statistical validity of a given correlation function fit and derived flow vector, prior to outputting the final vector map. For each temporal lag, τ, the radii $\omega_{x,y}(\tau)$ are compared to a set threshold value in order to remove abnormally broad correlation functions. These can occur if a macroscopic object, larger than the optical point spread function, migrates within the given ROI-TOI. We used a threshold value of 10 pixels, since objects larger than this size will create a broadened correlation function.

If sequential fitting (at different τ) of the 2D Gaussian does not yield at least two values of valid τ lags (criterion above) that can be used to fit linearly $v_{x,y}\tau$ vs. τ, then this ROI-TOI vector is assigned not-a-number (NaN) value and not included in the vector map. The vectors that pass these two criteria are compared to 8 of their nearest neighbours (neighbourhood). The mean orientation and the standard deviation for a given neighbourhood of vectors is calculated. The magnitude and orientation of a given vector is compared to the mean value of the neighbourhood. If its value is outside of the 3 standard deviations range from the mean, the vector is assigned NaN value. This ensures that the vectors that passed the other filtering criteria conform to the neighbourhood range of orientation. The final vector maps display the vectors only for regions where all filtering criteria were met.

Supplementary References

1. Brown CM*, et al.* Probing the integrin-actin linkage using high-resolution protein velocity mapping. *J Cell Sci* **119**, 5204-5214 (2006).

2. Hebert B, Costantino S, Wiseman PW. Spatiotemporal Image Correlation Spectroscopy (STICS) Theory, Verification, and Application to Protein Velocity Mapping in Living CHO Cells. *Biophysical journal* **88**, 3601-3614 (2005).

3. Toplak T, Pandzic E, Chen L, Vicente-Manzanares M, Horwitz AR, Wiseman PW. STICCS reveals matrix-dependent adhesion slipping and gripping in migrating cells. *Biophysical journal* **103**, 1672-1682 (2012).
